# Adolescents cholesterol passport: a universal pediatric lipid screening tool to combat atherosclerosis—the world's deadliest scourge

**DOI:** 10.3389/fped.2023.1228483

**Published:** 2023-06-28

**Authors:** Andrew O. Agbaje

**Affiliations:** ^1^ Institute of Public Health and Clinical Nutrition, School of Medicine, Faculty of Health Sciences, University of Eastern Finland, Kuopio, Finland; ^2^ Children’s Health and Exercise Research Centre, Department of Public Health and Sports Sciences, Faculty of Health and Life Sciences, University of Exeter, Exeter, United Kingdom

**Keywords:** dyslipidaemia, pediatrics, prevention and health promotion, screening, atherosclerosis, cardiovascular disease, global health, preventive cardiology

*“Now four men who were lepers were at the entrance of the city’s gate; and they said to one another, **Why do we sit here until we die?** If we say, we will enter the city, then the famine is in the city, and we shall die there; and if we sit still here, we die also. So now come, let us go over to the army of the Syrians. If they spare us alive, we shall live; and if they kill us, we shall but die.”*

– 2 Kings 7:3,4 AMPC

As the world emerges from a brutal combat with Covid-19, some dire lessons are being learnt, such as the danger of inaction or delayed action. A rapid galvanization of every facet of the society was necessary to halt the infectious disease's sophisticated spread and mutation. However, we have been somewhat double-minded concerning a global fight in preventing the world's deadliest scourge—atherosclerotic cardiovascular disease and its sequelae (1). The concept of prevention rather than cure appears a furlough considering the millions of dollars spent annually on the treatment of the consequences of atherosclerosis in the adult population, with largely unsatisfactory outcomes (2).

Over the last two decades, substantive longitudinal evidence aptly summarized in a recent review revealed that elevated cholesterol from childhood is a strong predictor of atherosclerosis in mid-adulthood, irrespective of the means of assessing atherosclerosis, either via a coronary artery calcium score or an increase in carotid intima-media thickness (1). The consistent report supports causal inferences, irrespective of ethnicity, race, sex, socio-economic, and educational background (1). Nonetheless, gaps in knowledge still exist on likely sensitive or crucial ages when cholesterol has a particularly strong effect on the development of atherosclerosis in a general asymptomatic pediatric population (1). It was reported two decades ago that children with familial hypercholesterolemia showed signs of significant carotid intima-media thickness deviation at age 12 years (3). This indeed is a crucial age for intervention in children with familial hypercholesterolemia. Three years ago, a 20-year follow-up study of statin therapy in 214 children with familial hypercholesterolemia concluded that initiating statin therapy during childhood slowed the progression of carotid intima-media thickness and reduced the risk of cardiovascular disease in adulthood (4). Importantly, low-density lipoprotein cholesterol (LDL-c) was the targeted culprit in childhood familial hypercholesterolemia, and only twenty percent of the treated children had normal LDL-c at the end of the 20-year follow-up period (4). Although the success rate of such treatment in relation to the cost and study duration may be unknown, the significant reduction in cardiovascular events in the study population in relation to their untreated parents, suggests that early prevention rather than cure remains the gold standard for combating dyslipidemia-related atherosclerotic cardiovascular diseases. A few days ago, a simulated model showed that early treatment of the progression of familial hypercholesterolemia in 1,000 hypothetical 10-year-olds, would gain 2.53 quality-adjusted life years per person, at an additional cost of €23,365 ($25,468) (both discounted). The estimated return on investment for the detection and treatment program for familial hypercholesterolemia in children was €8.37 ($9.12) (5).

There is yet no consensus on general lipid screening among asymptomatic pediatric populations who subsequently form a major proportion of future cardiovascular disease patients during mid-adulthood (1, 6, 7). The US Preventive Services Task Force (USPSTF) that publishes recommendations about the effectiveness of specific preventive care services for patients without obvious related signs or symptoms concluded in 2016 that “current evidence is insufficient to assess the balance of benefits and harms of screening for lipid disorders in children and adolescents 20 years or younger” (6). Their conclusion was based on the lack of clinical trials that assessed the benefits and harms of combined screening and treatment programs for familial hypercholesterolemia in children and adolescents (6). However, 3 years after the USPSTF recommendation the New England Journal of Medicine (NEJM) published a report on the 20-year follow-up of statin treatment in children with familial hypercholesterolemia, as summarized above (4). Moreover, the USPSTF recommendation highlighted knowledge gaps in longitudinal research on adverse cholesterol levels, intermediate outcomes in childhood and adolescence, and premature myocardial infarction and stroke in adulthood (6).

A few months ago, a longitudinal study published in NEJM conducted among 38,589 children, mean age 11.8 years (49.7% male and 15.0% Black) with an average follow-up of 35 years, reported a hazard ratio for a fatal cardiovascular event in mid-adulthood of 1.30 (95% confidence, 1.14–1.47) per unit increase in the z-score for total cholesterol level in childhood (8). The hazard ratio for fatal or non-fatal cardiovascular events in adulthood attributed to childhood total cholesterol z-score was 1.31 (1.22–1.42) (8). In two adolescents with familial hypercholesterolemia aged 12 and 16 years, aggressive treatment of LDL-c resulted in a significant regression of atherosclerotic plaques within 6 months (9). These reports fill one of the knowledge gaps identified by the USPSTF 6 years ago (6).

A few months later, a longitudinal study on intermediate outcomes using repeated measures of carotid intima-media thickness among 1,799 asymptomatic adolescents followed up for 9 years with measures of total cholesterol, high-density lipoprotein cholesterol (HDL-c), LDL-c, triglycerides at ages 15, 17, and 24 years, respectively observed that 1 in 5 adolescents at age 17 years had elevated lipid and dyslipidemia (10). Moreover, 1 in 4 adolescents had elevated lipid and dyslipidemia at age 24 years, but only 1 in 1,000 had received treatment by age 17 years (10). The study concluded that elevated and dyslipidemia levels of total cholesterol, non-HDL-c, and very low HDL-c but not LDL-c were significantly associated with carotid intima-media thickness progression from adolescence through young adulthood (10).

Since randomized clinical trials are non-existent on the effect of lipid screening and intervention on intermediate outcomes in an asymptomatic pediatric population (6), the study modelled an intervention effect using longitudinal data and reported that simulated intervention at age 24 years was ineffective to attenuate and reverse carotid intima-media thickness progression (10). However, simulated intervention at age 17 years effectively neutralized and potentially regressed carotid intima-media thickness progression (10). This study observed a high prevalence of undiagnosed elevated lipid and dyslipidemia in an asymptomatic pediatric population. It also identified the crucial age (age 17 years) for significant carotid intima-media thickness deviation in an asymptomatic pediatric population without familial hypercholesterolemia, thereby providing evidence for future clinical trials and universal lipid screening recommendations (1, 6). Increasing levels of non-HDL cholesterol in asymptomatic adolescents and young adults is a strong risk factor for subclinical atherosclerosis and should be an important focus of primordial and primary prevention (10). Likewise, ≥30 mg/dl levels of lipoprotein(a), a genetically determined and causal risk factor for atherosclerosis (11), during ages 9 through 24 years has been shown to double the risk of atherosclerotic cardiovascular disease by the median of age 47 years (12).

In conclusion, as quoted above “why sit here until we die?” is a story from the bible that resonates with the morbid danger of inaction or delayed action. Four men at a leper's colony were faced with death from famine or death from an army that had sufficient food at their disposal. These men made a fabulous gamble not to die of inaction but die honorably in search of food. Fortunately, they did not die, because they met food in abundance since the army already fled their base. In the same vein, atherosclerotic cardiovascular disease progression begins in early life and could cause premature death as early as age 40 years (8). Do we still need to wait until age 40 years for lipid screening? How many opportunities are missed before 40 years? What about the likelihood of failed or ineffective treatment as early as age 24 years, since it took 20 years to normalize lipid level with only a twenty percent success rate in patients with familial hypercholesterolemia? (4, 10)

The American Academy of Pediatrics and the National Heart, Lung, and Blood Institute recommendations for dyslipidemia screening have been put forward for children since 2011/2012 (13). In 2017, it was recommended that risk assessment for dyslipidemia should occur once between 9 and 11 years of age, and once between 17 and 21 years of age, which has now been updated to early risk assessment beginning at age 2 years (24 months) (14). Unfortunately, these periodicity schedules with recommendations for preventive pediatric health care dyslipidemia screenings have not been widely put into practice (14, 15). Inadequate health staff/resources for the care of children and adolescents at risk of dyslipidemia have plagued the implementation of screening, and the impact of socio-economic determinants of health and policy on the cardiovascular health of the pediatric population remains a critical unresolved challenge (15). To address some of these challenges, preventive medicine could be integrated into undergraduate medical training schools and pediatric residency programs. In 2019, the American College of Cardiology Council published a perspective for establishing a dedicated preventive cardiology subspecialty to train future clinicians and outlined possible paths to professional certification (16). Pediatric generalists as well as subspecialists including gastrointestinal, endocrinology, rheumatology, renal, and cardiology sub-specialists could also play a bigger role in ensuring dyslipidemia screening.

Just as the world fought Covid-19 and smallpox, could we combat dyslipidemia-related atherosclerotic cardiovascular disease by proposing an “adolescent’s cholesterol passport”? Immunization or vaccination card was instrumental to the global coverage of vaccines for infectious diseases such as polio, and evaluating adherence to immunization schedule from birth (17). This strategy was integral to the eradication of smallpox (17). Employing the same strategy as the Covid-19 vaccine passport and immunization card, the adolescent's cholesterol passport could be a universal screening tool for certifying cholesterol assessment by the end of teenage years. This could be encouraged before the first driver's license is granted, or as a prerequisite for completing high school studies, with a subsequent re-assessment once every 10 years. Every institution such as health, education, religious, socio-cultural, sports and athletics, and transport agencies, as well as local, regional, and national government agencies, should be responsible for raising awareness of the risk of dyslipidemia. Accountability measures for care providers and families should be enshrined in legislation, and strategies for public awareness and health education should be put in place.

A recent review that summarized clinical evidence with a strong appeal to preventing atherosclerotic diseases in the general population concluded that “*there is that fierce urgency of now: every day of delay means more people losing arterial health, with all the tragic consequences that result. We have the means; do we have the will?*” (2). Thankfully, the European Atherosclerosis Society in a consensus statement in tandem with two recently published papers highlighted challenging priorities such as improving education, early diagnosis, and treatment, as well as addressing inequities in access to all therapies, and advocated for pediatric universal lipid screening that will also improve the detection (7, 18, 19). Moreover, clinical trials and interventions among young participants free of familial hypercholesterolemia that would establish the simulated evidence are urgently warranted (5, 10). The United Nations Declaration of Human Rights that everyone has the right to life could as well be extended to include the right to an “elevated cholesterol-free life”.

A future generation beckons on us to summon the courage to combat dyslipidemia-related atherosclerosis by first establishing and implementing a universal pediatric lipid screening. ***Why do we sit here until we die?***
